# A blood-based prognostic biomarker in IBD

**DOI:** 10.1136/gutjnl-2019-318343

**Published:** 2019-04-27

**Authors:** Daniele Biasci, James C Lee, Nurulamin M Noor, Diana R Pombal, Monica Hou, Nina Lewis, Tariq Ahmad, Ailsa Hart, Miles Parkes, Eoin F McKinney, Paul A Lyons, Kenneth G C Smith

**Affiliations:** 1 Department of Medicine, University of Cambridge, Cambridge, UK; 2 Cambridge Institute of Therapeutic Immunology & Infectious Disease, University of Cambridge, Cambridge, UK; 3 PredictImmune Ltd, Cambridge, UK; 4 Nottingham University Hospitals NHS Trust, Nottingham, UK; 5 University of Exeter Medical School, Exeter, UK; 6 St Mark’s Hospital, London, UK; 7 Antigen Presentation Research Group, Imperial College, London, UK

**Keywords:** crohn’s disease, ulcerative colitis, gene expression, Ibd clinical, Ibd basic besearch

## Abstract

**Objective:**

We have previously described a prognostic transcriptional signature in CD8 T cells that separates patients with IBD into two phenotypically distinct subgroups, termed IBD1 and IBD2. Here we sought to develop a blood-based test that could identify these subgroups without cell separation, and thus be suitable for clinical use in Crohn’s disease (CD) and ulcerative colitis (UC).

**Design:**

Patients with active IBD were recruited before treatment. Transcriptomic analyses were performed on purified CD8 T cells and/or whole blood. Phenotype data were collected prospectively. IBD1/IBD2 patient subgroups were identified by consensus clustering of CD8 T cell transcriptomes. In a training cohort, machine learning was used to identify groups of genes (‘classifiers’) whose differential expression in whole blood recreated the IBD1/IBD2 subgroups. Genes from the best classifiers were quantitative (q)PCR optimised, and further machine learning was used to identify the optimal qPCR classifier, which was locked down for further testing. Independent validation was sought in separate cohorts of patients with CD (n=66) and UC (n=57).

**Results:**

In both validation cohorts, a 17-gene qPCR-based classifier stratified patients into two distinct subgroups. Irrespective of the underlying diagnosis, IBDhi patients (analogous to the poor prognosis IBD1 subgroup) experienced significantly more aggressive disease than IBDlo patients (analogous to IBD2), with earlier need for treatment escalation (hazard ratio=2.65 (CD), 3.12 (UC)) and more escalations over time (for multiple escalations within 18 months: sensitivity=72.7% (CD), 100% (UC); negative predictive value=90.9% (CD), 100% (UC)).

**Conclusion:**

This is the first validated prognostic biomarker that can predict prognosis in newly diagnosed patients with IBD and represents a step towards personalised therapy.

**Significance of this study**

**What is already known about this subject?**

- The course of Crohn’s disease (CD) and UC varies considerably between patients, but reliable prognostic markers are not available in clinical practice. This hinders disease management because treatment approaches that would be optimal for patients with indolent disease—characterised by infrequent flare-ups that can be readily controlled by first-line therapy— will inevitably undertreat those with progressive disease. Conversely, strategies that would appropriately control frequently relapsing, progressive disease will expose patients with more quiescent disease to the risks and side effects of unnecessary treatment. We have previously described a CD8 T cell gene expression signature that corresponds to differences in T cell exhaustion, is detectable during active untreated disease (including at diagnosis) and predicts disease course in both UC and CD. However, the need for cell separation and microarray-based gene expression analysis would make this difficult to translate to clinical practice.

**What are the new findings?**

- We have developed, optimised and independently validated a whole blood qPCR-based classifier—designed to identify the IBD1 and IBD2 patient subgroups—that can reliably predict prognosis in patients with CD or UC from diagnosis without the need for cell separation. We also present a detailed phenotypic update on the disease course experienced by patients in either the IBD1/IBDhi or IBD2/IBDlo subgroups, incorporating both expanded patient cohorts and substantially longer follow-up. This affords new insights into the spectrum of therapies that are differentially required in these patient subgroups and reinforces their association with disease prognosis.

**Significance of this study**

**How might it impact on clinical practice in the foreseeable future?**

- The qPCR-based classifier has performance characteristics that compare favourably with prognostic biomarkers currently in use in oncology and should be sufficient to guide therapy from diagnosis in patients with CD or UC. This represents an important step towards personalised therapy in IBD.

## Introduction

In recent years, there has been a growing realisation that the future of IBD management needs to incorporate a personalised approach to therapy, in which the right treatment can be given to the right patient at the right time.^1^ This now represents a key goal in IBD and was recently named as one of the most important research priorities by the James Lind Alliance priority-setting partnership^2^—a group of clinicians, patients and other stakeholders who sought to identify important areas of unmet need. In truth, this ambition is shared across many disease areas, motivated by developments in oncology where personalised therapy has been achieved using biomarkers that can accurately predict cancer outcome and response to therapy.^3 4^ The potential advantages of personalised medicine in IBD are clear. First, this would anticipate the marked variability in prognosis that occurs between patients^5 6^ and which means that ‘one-size-fits-all’ approaches cannot optimally treat everyone (either because they are ineffective in some or unnecessarily risky in others). Second, it would enable clinicians to better use the growing armamentarium of IBD therapies to improve clinical outcomes.^7^ For example, it is well recognised that early use of combination therapy (anti-tumour necrosis factor (TNF)α monoclonal antibodies and an immunomodulator) is one of the most effective treatments in CD,^8^ particularly when given early in the disease course,^9 10^ but that indiscriminate use of this strategy would be prohibitively expensive and expose many patients to side effects of drugs that they do not require. Unfortunately, in IBD—as in most autoimmune and inflammatory diseases—biomarkers that can reliably predict disease course from diagnosis are not available, precluding the delivery of personalised therapy.

We have previously reported that hypothesis-free inspection of CD8 T cell gene expression data from patients with active, untreated autoimmune disease can identify thousands of genes whose differential expression defines two distinct patient subgroups.^11 12^ Notably, these subgroups were not detectable using unsupervised analysis of unseparated peripheral blood mononuclear cells (PBMCs) from the same patients.^11 12^ In all of the diseases studied, including CD and UC, these subgroups were clinically indistinguishable at enrolment, but patients within them subsequently experienced contrasting disease courses, characterised by differences in the time to first relapse and the number of treatment escalations required over time.^11 12^ More recent work has ascribed the gene signature to inter-patient differences in T cell exhaustion^13^: the phenomenon by which effector T cells progressively lose their ability to respond to target antigens. T cell exhaustion was originally reported as a consequence of chronic viral infection^14^ but is now recognised to occur with persistent auto-antigens.^13 15^ Consistent with being less able to respond to disease-related antigens, patients with more T cell exhaustion had a better prognosis, characterised by a longer time to disease relapse and fewer flares over time.^13^

Here, we describe how we have developed, optimised and independently validated a whole blood biomarker—designed to identify the IBD1/IBD2 subgroups—that can predict the course of UC and CD from diagnosis. Additionally, we present a detailed phenotypic update regarding the clinical consequences of being in the IBD1 (exhaustion low) or IBD2 (exhaustion high) subgroups.

## Materials and methods

### Patient recruitment (training cohort for biomarker discovery and CD8 T cell cohort—Cambridge)

Patients with active CD and UC, who were not receiving concomitant corticosteroids, immunomodulators or biological therapy, were recruited from a specialist IBD clinic at Addenbrooke’s hospital, Cambridge, before commencing treatment. A stable dose of topical or oral 5-ASA was permitted if patients had been diagnosed previously. All subjects were recruited between 2008 and 2014 and were aged 18 years or older. Most (86/118) were recruited at the time of diagnosis. All patients were diagnosed with CD or UC based on standard endoscopic, histological and radiological criteria and were treated in accordance with national and international guidelines using a conventional step-up strategy within the UK National Health Service. Disease activity was assessed by considering symptoms, clinical signs, blood tests (C reactive protein, haemoglobin and albumin), stool markers (calprotectin) and endoscopic assessment where indicated. To be enrolled, patients had to have active disease confirmed by one or more objective marker (raised CRP, raised calprotectin or endoscopic evidence of active disease) in addition to active symptoms and/or signs (table 1). Clinicians were blinded to the biomarker results. Detailed phenotype data were collected prospectively. All participants provided written informed consent.

**Table 1:** Baseline patient characteristics in CD8 T cell cohort

	CD			UC		
	IBD1 (n=33)	IBD2 (n=33)	P value	IBD1 (n=24)	IBD2 (n=28)	P value
Age (years)	30.3 (25.3–36.1)	30.3 (23.2–38.7)	0.98	43.8 (30.9–50.4)	40.5 (29.1–54.0)	0.92
Gender (% male)	14 (42.4%)	13 (39.4%)	1.00	13 (54.2%)	13 (46.4%)	0.78
Current smoker	10 (28.6%)	12 (33.3%)	0.79	1 (4.2%)	0 (0%)	0.46
Newly diagnosed	27 (81.8%)	24 (72.7%)	0.56	15 (62.5%)	20 (71.4%)	0.56
Disease duration (years)	0.0 (0.0–0.0)	0.0 (0.0–0.0)	0.78	0.0 (0.0–2.2)	0.0 (0.0–3.6)	0.76
Haemoglobin (g/L)	12.5 (11.7–13.3)	13.1 (11.8–13.6)	0.63	14.0 (12.8–14.4)	13.0 (12.3–14.6)	0.26
CRP (mg/L)	26 (16–39)	25 (10–59)	0.60	6 (3–23)	4 (2–21)	0.26
Albumin (g/L)	35 (32–37)	37 (34–39)	0.14	39 (37–41)	39 (37–41)	0.96
Disease distribution:						
CD – L1 (ileal)	9 (27.3%)	13 (39.4%)	0.43	–	–	–
CD – L2 (ileocolonic)	11 (33.3%)	9 (27.3%)	0.79	–	–	–
CD – L3 (colonic)	13 (39.4%)	11 (33.3%)	0.80	–	–	–
CD – L4 (upper GI)	2 (6.1%)	3 (9.1%)	1.00	–	–	–
Perianal	6 (18.2%)	3 (9.1%)	0.48	–	–	–
UC – E1 (proctitis)	–	–	–	5 (20.8%)	8 (28.6%)	0.75
UC – E2 (left sided)	–	–	–	9 (37.5%)	11 (39.3%)	1.00
UC – E3 (extensive)	–	–	–	10 (41.7%)	9 (32.1%)	0.57
Prospective follow-up (years)	4.9 (3.6–7.4)	5.3 (4.3–8.3)	0.24	5.6 (3.6–7.1)	5.5 (2.4–8.4)	0.54

Data shown in parentheses indicate median (IQR) for continuous variables or percentages for dichotomous variables. Statistical significance was calculated using a Mann-Whitney test (two tailed) for continuous variables and Fisher’s exact test (two tailed) for dichotomous variables. Disease distribution was classified according to the Montreal Classification.^27^

CD, Crohn’s disease; CRP, C reactive protein.

### Sample preparation

A 110 mL venous blood sample was taken from patients at enrolment. PBMCs were immediately extracted and CD8 T cells were positively selected, as described previously.^16^ Following purification, cells were lysed and lysates stored at −80°C. RNA was subsequently extracted using RNeasy Mini Kits (Qiagen) and quantified using a NanoDrop1000 Spectrophotometer (ThermoFisher). Of the total blood draw, 2.5 mL were collected into a PAXgene Blood RNA tube IVD (PreAnalytix), which was stored at −80°C. Whole blood RNA was subsequently extracted using a PAXgene 96 Blood RNA kit (PreAnalytix) according to the manufacturer’s instructions.

### Microarray processing and analysis

Following assessment of RNA quality (2100 Bioanalyzer, Agilent Technologies), 200 ng RNA was processed for hybridisation onto Affymetrix Human Gene 1.0 ST microarrays (CD8 T cell samples, n=118) or Affymetrix Human Gene 2.0 ST microarrays (whole blood samples, n=69) according to the manufacturer’s instructions. Raw data were preprocessed (background corrected, normalised, quality checked and batch normalised) using Bioconductor packages (http://www.bioconductor.org/) in R (http://www.r-project.org/): *affy*,^17^ *vsn*,^18^ *arrayQualityMetrics* 19 and *sva*.^20^ For CD8 T cell data, unsupervised consensus clustering was performed to identify the IBD1/IBD2 subgroups, as previously described.^12^ Of note, IBD1/IBD2 status was not included as a covariate in the batch normalisation of whole blood samples to reduce any downward bias in estimating the generalisation error during leave-one-out cross-validation (LOOCV).

### Biomarker development

Following preprocessing, a statistical (machine) learning method—logistic regression with an adaptive Elastic-Net penalty^21^ —was applied to the whole blood transcriptomic data to identify genes that could be used to calculate the probability of an individual belonging to the IBD1/IBD2 subgroups. Penalised regression methods are a useful tool to regularise models, and thus control overfitting, during biomarker discovery.^22^ The adaptive Elastic-Net method in particular combines the strengths of the ridge penalty and the adaptively weighted lasso shrinkage penalty and can address the technical challenges in these data.^21^ These were: high dimensionality (ie, number of samples is substantially smaller than number of genes), multicollinearity (ie, expression of many genes is correlated, with the need to avoid selecting multiple correlated genes in the model) and requirement for a sparse and interpretable model (ie, need for a limited number of genes in a classifier in which the contribution of each can be interpreted). The initial model was determined using a classic Elastic-Net (implemented in the *gcdnet* package^23^ in R) followed by adaptive Elastic-Net training using equations reported in the original description of the method.^21^ In brief, the optimal classification rule to identify the IBD1/IBD2 subgroups was learnt from the whole blood microarray data by defining many different combinations of model hyperparameters, which were then used to fit a corresponding number of candidate models (2100) to the whole blood expression data. Model selection was performed using the Bayesian Information Criterion (BIC), where the highest BIC corresponds to the best model (online supplementary table 1). BIC was defined as:

10.1136/gutjnl-2019-318343.supp1 Supplementary data

(n) $$BIC=-\ln\left(\hat{L}\right)-k\cdot ln\left(n\right)$$

where k=degrees of freedom (the number of genes incorporated), n=number of samples and (L)=log likelihood function for the model. The generalisation error of the selected model was estimated using nested LOOCV.^24^

### qPCR classifier development

A list of 39 candidate and 3 reference genes was taken forward to qPCR classifier development using TaqMan gene expression assays (online supplementary table 2). Following reverse transcription of whole blood RNA, qPCR was performed in triplicate using a Roche LightCycler 480, and transcript abundance was calculated using the ΔΔCT method, based on the mean of technical replicates. The correlation between microarray and qPCR expression values was then used to filter the candidate gene list (six were removed due to poor correlation). This resulted in a dataset containing expression values for 33 candidate and 3 reference genes from 69 samples. Following normalisation by feature standardisation, an identical penalised regression strategy was applied to this qPCR dataset to identify an optimal classification model comprising 16 informative and 2 reference genes. To refine this model for use on unscaled data, a prerequisite for use in a clinical setting, an additional round of penalised logistic regression was applied using the *cvglmnet* function in the *glmnet* package^22^ in R. This uses iterative cross-validation undertaken concurrently to facilitate automatic identification of the optimal, or most regularised, model (using accuracy of IBD1/IBD2 classification as a performance metric). This identified a 17-gene model (15 informative and 2 reference genes) with an error within 1 SE of the minimum mean cross-validated error, which was considered the most regularised (as recommended by the authors of this approach^22^). This 17-gene classifier was ‘locked-down’ so that no further changes could be made and was then tested in the validation cohorts. Patients in the qPCR subgroup analogous to IBD1 were termed ‘IBDhi’ and patients in the subgroup analogous to IBD2 were termed ‘IBDlo’.

10.1136/gutjnl-2019-318343.supp2 Supplementary data

### Validation cohorts

One hundred and twenty-three patients with active IBD (66 CD, 57 UC) were recruited before commencing treatment from specialist clinics in four UK teaching hospitals (in Cambridge, Nottingham, Exeter and London). All subjects were recruited between 2009 and 2017 and were aged 18 years or older. The median follow-up was 1.9 years (IQR: 1.3–3.2 years). Of these patients, 115 (93%) were newly diagnosed (61 CD, 54 UC). Prospective follow-up data were collected for all patients, who were treated at the discretion of their gastroenterologists in accordance with national and international guidelines. Clinicians were blinded to gene expression analyses. From each patient, a 2.5 mL venous blood sample was collected into a PAXgene Blood RNA tube IVD (PreAnalytix), which was stored at −80°C. RNA was subsequently extracted, quantified and quality checked as described above. qPCR was performed for the 15 informative and 2 reference genes within the optimal classifier using Research-Use-Only PredictSURE IBD kits (PredictImmune) to determine whether patients were IBDhi or IBDlo. The clinical course experienced by the IBDhi and IBDlo subgroups was compared using prospectively collected phenotype data. Importantly, the phenotyping collection was blinded to the classifier designation and vice versa. All participants provided written informed consent.

### Statistical analysis

Statistical tests performed during microarray analysis or machine learning are described in the relevant sections. Survival analyses for time-to-first-treatment-escalation were performed using a log-rank test. Comparison of the number of treatment escalations was performed using a Mann-Whitney test (two tailed for CD8 T cell analyses and one tailed for validation cohort analyses). Comparison of the clinical and laboratory data in IBD1/IBD2 patients was performed using Fisher’s test for dichotomous variables or Mann-Whitney test for continuous variables (two tailed). The α value for these analyses was 0.05. All statistical analyses and reporting were performed in accordance with Strengthening the Reporting of Observational Studies in Epidemiology guidelines.^25^

## Results

### Whole blood classifier development

**Figure 1:**
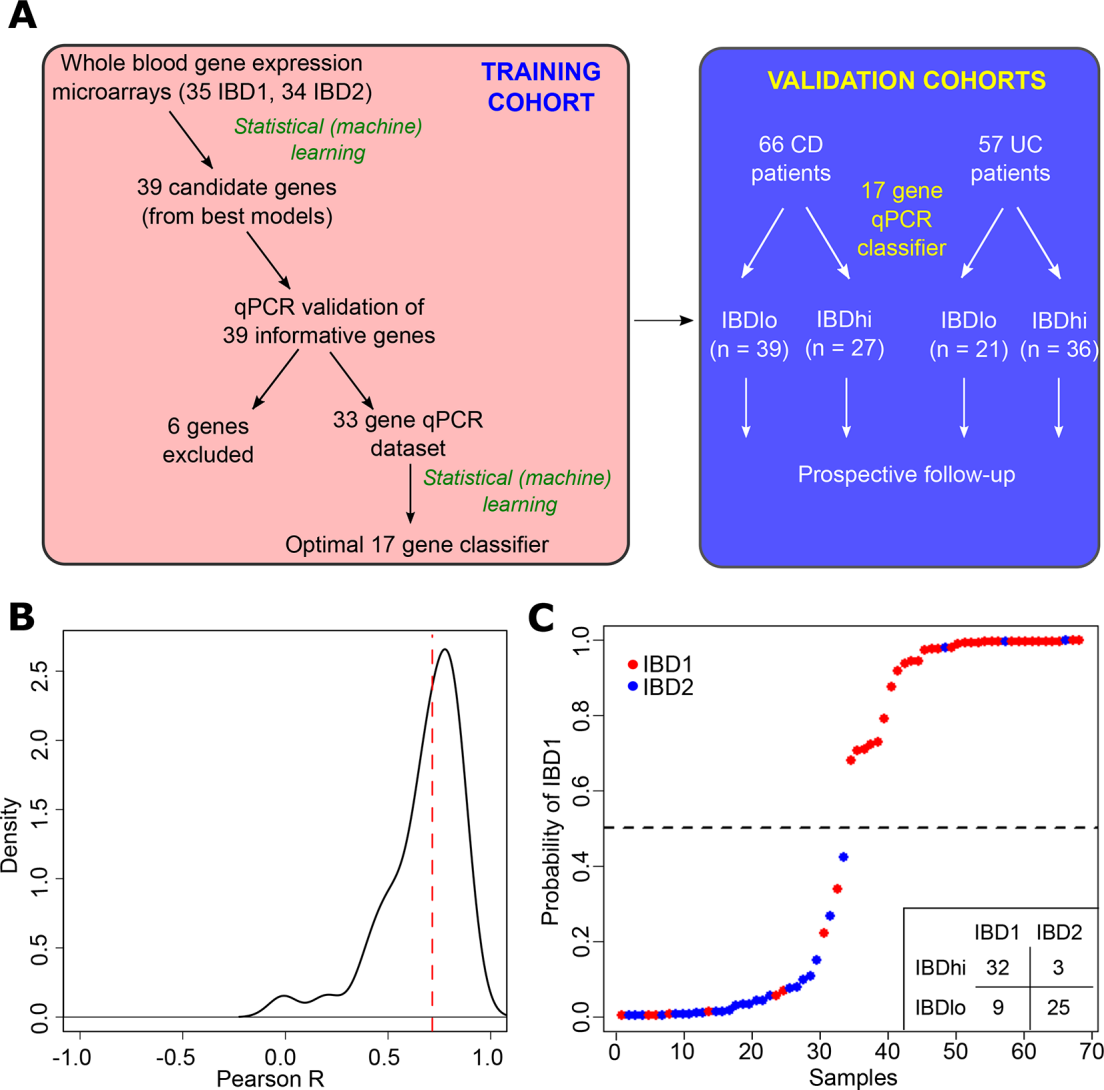
Development of a qPCR-based whole blood prognostic biomarker. (A) Schematic depicting the workflow for the development, optimisation and validation of the whole blood qPCR-based classifier with separate training and validation cohorts. (B) Distribution of correlation coefficients between microarray and qPCR-based measurements of gene expression for 39 genes. (C) Confidence of assignments to IBD1 and IBD2 subgroups in the training cohort using the qPCR classifier (15 informative and 2 reference genes). Colours indicate actual IBD1/IBD2 assignments based on CD8 T cell transcriptomic analysis (red=IBD1, blue=IBD2). Inset summary table depicts results using 0.5 cut-off for group assignment. CD, Crohn’s disease.

We have previously reported that a prognostic biomarker based on IBD1/IBD2 subgroup membership would represent a useful clinical tool, given its performance characteristics.^12^ Nonetheless, it is clear that any assay that requires CD8 T cell purification and microarray analysis would be difficult to translate to clinical practice. For this reason, we investigated whether we could identify the same patient subgroups using whole blood, without the need for cell separation (figure 1A). To do this, we first defined a training cohort of 69 patients (39 CD, 30 UC; 35 IBD1, 34 IBD2) for whom we had both CD8 T cell transcriptomic data and a whole blood PAXgene Blood RNA sample (the latter taken at the same time as the CD8 T cell sample). Fifty of these patients were in our original report of IBD1/IBD2^12^ and 19 were recruited subsequently. RNA was extracted from PAXgene Blood RNA tubes, and genome-wide gene expression was measured by microarray (Affymetrix Human Gene 2.0 ST arrays). The resulting raw data were preprocessed to create a normalised dataset that could be used for classifier development (Materials and methods). To identify a whole blood classifier, we used a machine learning method (logistic regression with adaptive Elastic-Net penalisation^21^) to identify models comprising the smallest number of most predictive genes with least redundancy. A series of potential models were produced (online supplementary table 1) of which the optimal model comprised 12 genes and resulted in accurate identification of the IBD1/IBD2 subgroups (p=1.6×10^−7^ for comparison with a ‘dummy’ classifier using a binomial distribution of samples). The generalisation error for this model was estimated using LOOCV (accuracy=0.81, 95% CI 0.70 to 0.90).

### qPCR classifier development and optimisation

To translate this result into a clinically useful tool, we examined the top models and selected 39 candidate genes and 3 reference genes for qPCR optimisation (figure 1A, online supplementary table 2, Materials and methods). Of the candidate genes, 12 were members of the optimal microarray-based classifier, 6 were highly correlated with genes in the optimal classifier and 21 were selected from adaptive Elastic-Net models with lower BIC (online supplementary table 2). Genes that showed poor correlation with microarray data were excluded (n=6, figure 1A,B). Using qPCR data, we then applied a similar statistical learning strategy (Materials and methods) to identify the optimal classifier (15 informative and 2 reference genes; figure 1C, online supplementary table 3), which was locked down for further testing.

10.1136/gutjnl-2019-318343.supp3 Supplementary data

### qPCR classifier validation

**Figure 2:**
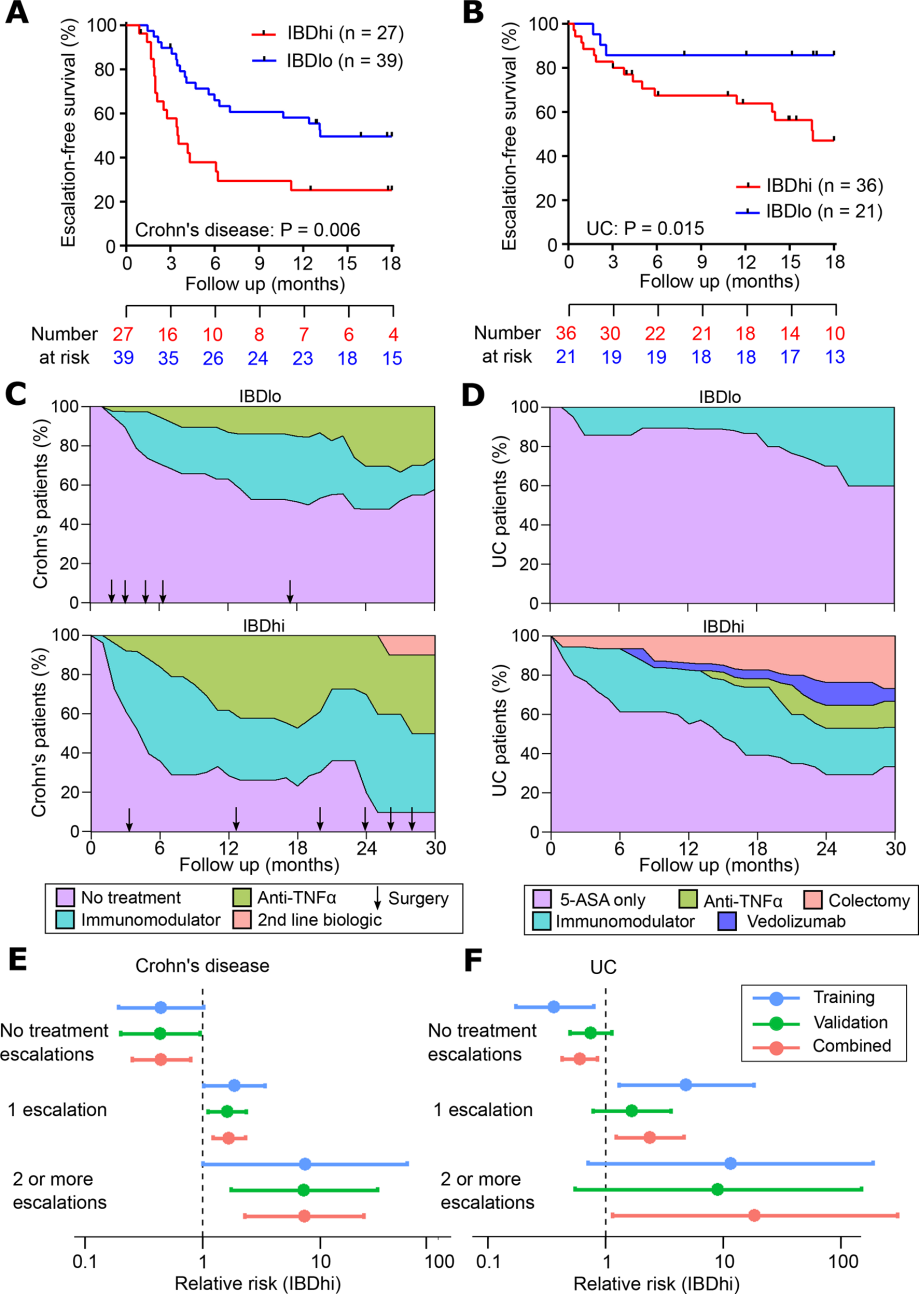
Validation of qPCR-based classifier in independent cohorts. (A and B) Kaplan-Meier plots of escalation-free survival for the CD validation cohort (A; n=66) and the UC validation cohort (B; n=57) as stratified by the IBDhi (IBD1 equivalent) and IBDlo (IBD2 equivalent) patient subgroups. Data are censored at 18 months. Statistical significance assessed by log-rank test. (C and D) Stacked density plots demonstrating the maximum medical therapy that was required during the first 2.5 years’ prospective follow-up of the IBDhi and IBDlo subgroups in CD (C) and UC (D). Treatments were plotted hierarchically (no treatment<immunomodulator<anti-TNFα<second-line biologicals (vedolizumab or ustekinumab) in CD and 5-ASA only<immunomodulator<anti-TNFα<vedolizumab < colectomy in UC). Arrows represent episodes of surgery that were required for CD patients at the indicated timepoints. Data are censored accordingly to length of follow-up so that the denominator is the total available cohort at each timepoint. (E and F) Forest plots of the relative risk (IBDhi vs IBDlo) of requiring no treatment escalations, one treatment escalation or two or more treatment escalations within the first 18 months after enrolment for patients with CD (E) and patients with UC (F). Relative risk is with respect to the IBDhi subgroup in each disease and is presented separately for the training cohort, validation cohort and combined cohorts. Error bars indicate 95% CIs. CD, Crohn’s disease.

A critical step in the development of any new biomarker is independent validation, in which the assay can be tested on samples that were not included in the discovery phase. This facilitates an assessment of whether the model will generalise to populations other than the one on which it was developed (figure 1A) and provides a more accurate estimate of the true performance characteristics of the assay. We therefore tested the qPCR classifier in the validation cohorts of patients with CD and UC. When applied to these independent samples, the classification algorithm assigned every patient into either the ‘IBDhi’ (analogous to IBD1) or ‘IBDlo’ (analogous to IBD2) subgroup. In both the CD and UC validation cohorts, patients in the IBDhi and IBDlo subgroups experienced very different disease courses. Patients in the IBDhi subgroup had consistently more aggressive disease, which was characterised by the need to escalate treatment earlier (with immunomodulators, biological therapies or surgery) and more frequently than for patients in the IBDlo subgroup (figure 2A–F). In the CD validation cohort, the HR for the difference in time to first escalation was 2.65 (95% CI 1.32 to 5.34; p=0.006) and in the UC validation cohort this HR was 3.12 (95% CI 1.25 to 7.72; p=0.015) (figure 2A,B). Moreover, irrespective of the underlying disease, IBDhi patients experienced a disease course that necessitated more potent therapies to achieve disease remission (figure 2C,D). The sensitivity and specificity for predicting the need for multiple escalations within the first 18 months were 72.7% and 73.2% in CD and 100% and 48% in UC. Of note, the relatively low specificity in UC reflects the lower treatment escalation rate observed (36 escalations in the UC validation cohort compared with 67 in the CD cohort) and thus while all of the UC patients who required multiple escalations were IBDhi, not all of the IBDhi patients had required multiple escalations within the first 18 months. Importantly, because this test would be used at diagnosis, negative prediction (ie, correctly identifying patients who do not need additional therapy) is more relevant,^26^ both to improve resource allocation and not miss a ‘window of opportunity’ to optimally treat patients with progressive disease. In these validation cohorts, the negative predictive value for predicting multiple escalations within the first 18 months was high: 90.9% in CD and 100% in UC (figure 2E,F). These results are particularly noteworthy given that the classifier was developed to predict IBD1/IBD2 subgroup membership (being directly assessed against this in the training cohort). In the validation cohorts, however, CD8 T cell transcriptomic data—and thus IBD1/IBD2 subgroup membership—was not available, and so the biomarker had to be assessed against the difference in prognosis that was observed in the IBD1/IBD2 subgroups. This is one step removed from how the classifier was developed and represents a more difficult benchmark but is ultimately what a prognostic biomarker would need to predict to be clinically useful.

To facilitate translation of this test to clinical practice, analytical validation was also performed to assess precision, limit of detection, linearity, input RNA range and freeze/thaw cycling for each gene’s qPCR assay and for the combined multianalyte-derived risk score (data not shown). The contribution of specific sources (eg, operator and batch) to the total assay variance was also assessed (data not shown). Together, these analytical and clinical validation data have resulted in a CE-marked assay that is ready for clinical use (PredictSURE IBD, PredictImmune).

### Clinical phenotype over time

It is clear that the phenotypic consequences of IBDhi/IBDlo subgroup membership mirror those observed in IBD1/IBD2 patients.^12^ However, due to their prospective collection, both of these cohorts had relatively limited follow-up (validation cohort: median 1.9 years; original CD8 T cell cohort manuscript^12^: median 1.6 years). To better understand the longer term consequences of being in the IBD1 (IBDhi) or IBD2 (IBDlo) subgroups, we examined the extended phenotyping data from all patients for whom CD8 T cell gene expression data were available. This cohort was now larger than previously reported^12^ (sample size increased from 67 to 118) and had substantially longer follow-up (median follow-up increased from 1.6 years to 5.3 years). These increases in cohort size and follow-up enabled us to perform a more detailed analysis of the clinical consequences of IBD1/IBD2 subgroup membership. Baseline patient characteristics are presented in table 1. Consistent with our previous findings, all patients could be readily classified into IBD1 or IBD2 based on CD8 T cell gene expression. There were no clinical characteristics at baseline that distinguished between these subgroups (table 1), and specifically there was no correlation between measures of inflammation and subgroup membership.

### Disease course in IBD1/IBD2 patients

**Figure 3:**
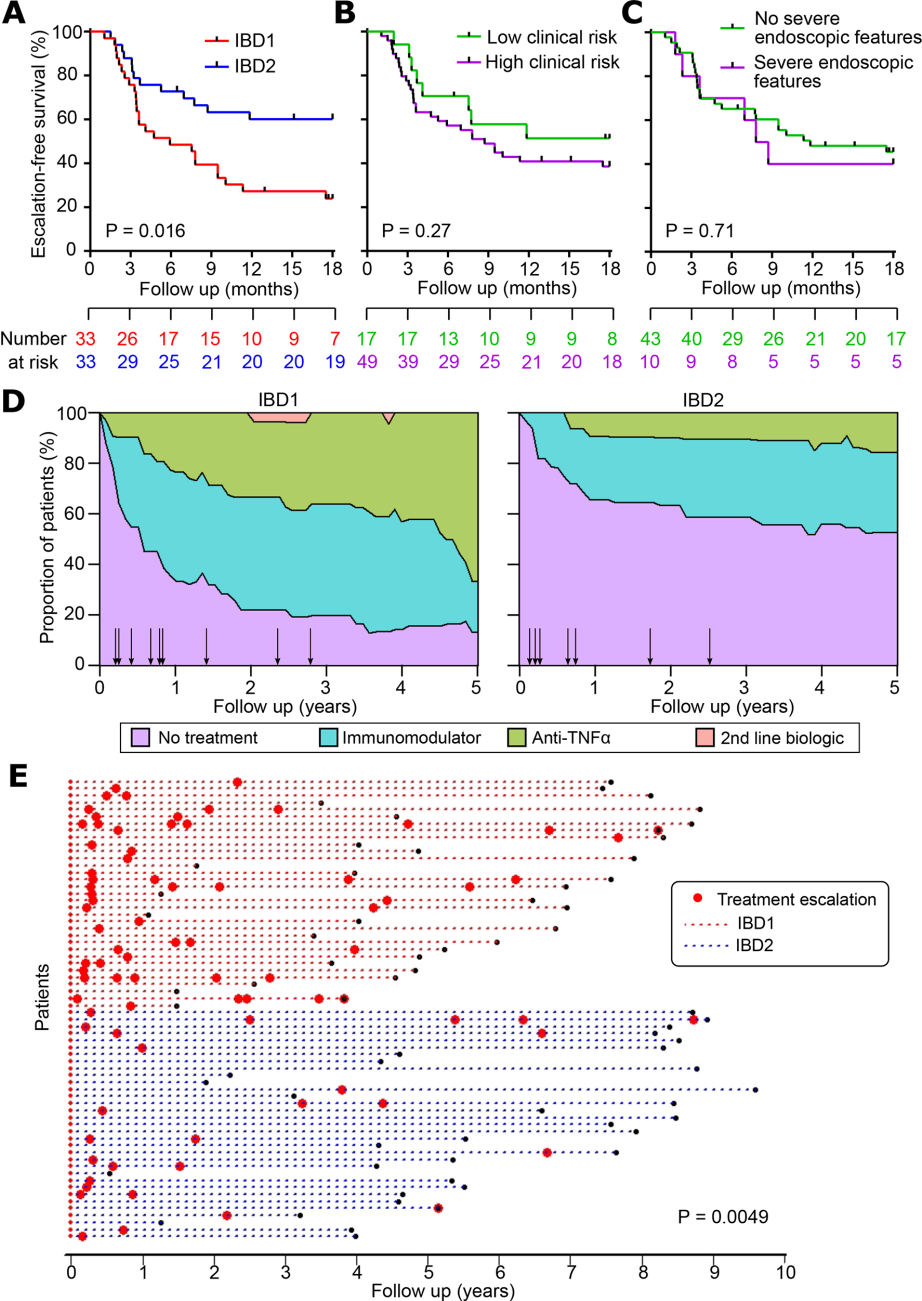
The clinical course of Crohn’s disease (CD) is different in IBD1 and IBD2 patients. (A) Kaplan-Meier plot of escalation-free survival for CD patients in the IBD1 and IBD2 subgroups. Data are censored at 18 months. Statistical significance assessed by log-rank test. (B and C) Kaplan-Meier plots in the same format as figure part A with patients subdivided according to clinical risk (high risk=2 or more of: age <40 years at diagnosis, early need for steroids and perianal disease; B) and presence of severe features at index endoscopy (deep and extensive ulceration in at least one colonic segment or endoscopist’s global assessment; C). (D) Stacked density plots demonstrating the maximum medical therapy that was required during 5 years’ prospective follow-up in the IBD1 and IBD2 subgroups. Treatments were plotted hierarchically (no treatment<immunomodulator<anti-TNFα<second-line biologicals (vedolizumab or ustekinumab)). Arrows represent episodes of surgery that were required at the indicated timepoints (of note one operation that is indicated in online supplementary table 4—in an IBD1 patient—occurred after 5 years and is not shown). Data are censored accordingly to length of follow-up so that the denominator is the total available cohort at each timepoint. (E) Disease course of individual CD patients (dotted lines). The colour of dotted lines reflects subgroup designation. Statistical significance was determined using a Mann-Whitney test.

*CD*: 66 patients with CD were recruited of whom 51 (77.3%) were newly diagnosed at enrolment. Thirty-three patients were in IBD1 and 33 in IBD2. Compared with patients in the IBD2 subgroup, IBD1 patients had a significantly shorter time to requiring a treatment escalation, as previously reported^12^ (figure 3A). Interestingly, neither clinical parameters (any two of: steroid requirement, age <40 years and perianal disease) nor severe endoscopic features (including deep and extensive ulceration in at least one colonic segment) were able to predict the need for early treatment escalation (figure 3B,C). Indeed, even if we attempted to incorporate these, or other, clinical features into a predictive classifier (using a Cox proportional hazards model), we found that none of them were able to improve the performance of the transcriptional classifier (data not shown).

10.1136/gutjnl-2019-318343.supp4 Supplementary data

IBD1 patients with CD also required significantly more treatment escalations over time due to persistently relapsing or chronically active disease (figure 3D,E). Indeed, in IBD1, the relative risk (RR) of requiring escalation to biologic therapy (excluding those who received biologic therapy due to immunomodulator intolerance) was 3.0 (12/33 IBD1 patients, 4/33 IBD2 patients) (online supplementary table 4). Likewise, the RR of not requiring any medical therapy in IBD1 was 0.53 (8/33 IBD1 patients, 15/33 IBD2 patients) (figure 3E, online supplementary table 4). Total surgery rates were not significantly different between the groups (10/33 IBD1, 7/33 IBD2), but the trend to a higher surgery rate in IBD1 mirrored that observed in the CD validation cohort (figure 2C) and so may simply reflect a lack of power to detect an effect. Notably, all of the patients who required a panproctocolectomy were in the IBD1 subgroup (online supplementary table 4). There were two deaths during follow-up: an IBD2 patient died from end-stage COPD and an IBD1 patient died from liver failure secondary to PSC.

**Figure 4:**
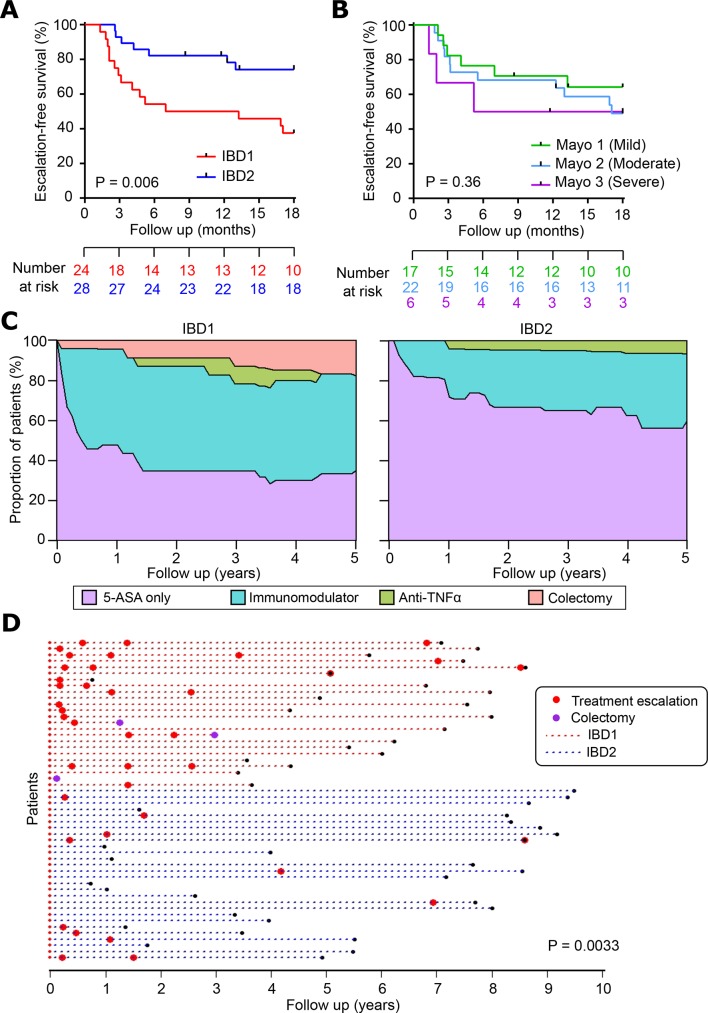
The clinical course of UC is different in IBD1 and IBD2 patients. (A) Kaplan-Meier plot of escalation-free survival for UC patients in the IBD1 and IBD2 subgroups. Data are censored at 18 months. Statistical significance assessed by log-rank test. (B) Kaplan-Meier plot in the same format as figure part A with patients subdivided according to endoscopic disease severity at index colonoscopy. P value calculated by comparing mild and severe cases. (C) Stacked density plots demonstrating the maximum medical therapy that was required during the first 5 years’ prospective follow-up in the IBD1 and IBD2 subgroups. Treatments were plotted hierarchically (5-ASA only<immunomodulator<anti-TNFα<vedolizumab<colectomy). Data are censored accordingly to length of follow-up so that the denominator is the total available cohort at each timepoint. (D) Disease course of individual UC patients (dotted lines). The colour of dotted lines reflects subgroup designation. Statistical significance was determined using a Mann-Whitney test.

*UC:* 52 patients with UC were recruited, of whom 35 (67.3%) were newly diagnosed. Twenty-four patients were in IBD1 and 28 in IBD2. As in the CD cohort, UC patients in the IBD1 subgroup experienced more aggressive disease with significantly earlier need for treatment escalation (figure 4A). Notably, endoscopic severity at baseline^28^ did not predict need for treatment escalation (figure 4B). Over time, IBD1 patients also required significantly more escalations due to recurrently active disease (figure 4C,D). There were several other similarities between the UC and CD cohorts, with the probability of not needing any treatment escalations in IBD1 UC patients being approximately half that of IBD2 UC patients (RR=0.45), and the RR of requiring escalation to biological therapy or colectomy in IBD1 being 4.08 (7/24 IBD1 patients, 2/28 IBD2 patients) (figure 4C,D, online supplementary table 4). Indeed, across all of the patient cohorts (CD8 T cell and whole blood) colectomies were only required in IBD1/IBDhi patients (7/56 IBD1 or IBDhi patients; 0/48 IBD2 or IBDlo patients, p=0.01, two-tailed Fisher’s test). There was one death during follow-up: an IBD1 patient who was due to start anti-TNFα therapy for chronically active disease died from a pulmonary embolism.

## Discussion

A major barrier to personalised medicine in CD and UC is the lack of biomarkers to guide treatment from diagnosis. Indeed, the performance requirements for a prognostic test mean that even though several parameters have been associated with prognosis in CD—including clinical features,^29^ serology^30^ and genetic variants^31^—none are sufficient to guide therapy in clinical practice. Accordingly, current treatment regimens tend to adopt a ‘one-size-fits-all’ approach, which cannot provide safe, effective and cost-efficient therapy for every patient. Here, we describe the development of a practical, whole blood assay that is in direct response to this unmet need. This assay is the first prognostic test in IBD that has validated performance characteristics that can support its use as a prognostic biomarker from diagnosis. Indeed, the performance characteristics of the qPCR classifier in both CD and UC are similar to that of existing gene expression-based in vitro diagnostic tests in oncology. For example, the HR for OncotypeDX, a gene expression diagnostic that predicts breast cancer recurrence,^32^ is 2.81 (95% CI 1.70 to 4.64).^33^ Importantly, the proven benefit of early aggressive therapy in IBD^9 10^ should only amplify the clinical benefit of using this assay to stratify patients at diagnosis, since IBDhi patients typically experience the sort of aggressive disease that should benefit most from early use of potent therapy. Collectively, these data support the early adoption of this assay in clinical practice, which should not be logistically difficult since a whole blood qPCR assay can be readily incorporated into standard laboratory protocols.

There are several limitations of this work. First, the study was non-interventional, and all patients were assessed and treated at the discretion of their gastroenterologists in accordance with national and international guidelines, rather than following a formal protocol. This, however, represents real-world practice and is the setting in which the test will ultimately be used. Second, because patients were recruited before induction therapy, we do not yet know how the biomarker would perform if treatment had already been started. Nonetheless, if induction therapy was underway, the biomarker could still be used if/when patients next re-flare, since the CD8 T cell signature is readily detectable during active disease.^13^ Clarifying the effect of concomitant therapy is the subject of ongoing work. Third, while the performance characteristics of this assay meet the requirements of a useful prognostic biomarker, we have not yet conducted an interventional study to confirm that stratifying therapy using this biomarker would improve clinical outcomes. For this reason, we have concurrently designed a biomarker-stratified trial^34^ to test whether this assay can deliver personalised therapy from diagnosis. This trial (Predicting outcomes for Crohn’s DIsease using a molecular biomarker; www.crohnsprofiletrial.com) is currently recruiting in the UK and represents one of the first biomarker-stratified trials in any inflammatory disease. It will assess the relative benefit of ‘Top Down’ therapy (anti-TNFα and an immunomodulator) over ‘Accelerated Step-Up’ therapy in IBDhi and IBDlo patients to determine whether the biomarker can accurately match patients to the most appropriate treatment for them, thereby improving outcomes by optimising disease control and minimising drug toxicity.

In summary, we have developed, optimised and validated a whole blood gene expression biomarker that can predict prognosis in patients with either CD or UC. This provides a rational basis for personalised therapy in IBD and represents an important step towards precision medicine for patients with CD or UC.
